# Cutaneous Adverse Reactions and Survival Outcomes of Advanced Melanoma Treated with Immune Checkpoint Inhibitors in an Academic Medical Centre in Singapore

**DOI:** 10.3390/diagnostics14151601

**Published:** 2024-07-25

**Authors:** Agnes Yeok-Loo Lim, Jason Yongsheng Chan, Choon Chiat Oh

**Affiliations:** 1 Department of Dermatology, Singapore General Hospital, Singapore 169608, Singapore; agnes.lim@mohh.com.sg; 2 Duke-NUS Medical School, Singapore 169857, Singapore; 3 Division of Medical Oncology, National Cancer Centre Singapore, Singapore 168583, Singapore

**Keywords:** melanoma, PD1 inhibitor, immunotherapy, cutaneous adverse reaction

## Abstract

Programmed cell death-1 (PD1) inhibitors, a form of immune checkpoint inhibitor, are efficacious for metastatic melanoma but are associated with cutaneous adverse reactions (CARs). Studies in Europe and North America showed that CARs are associated with an increased overall survival. However, studies from Asia showed mixed results. There is a paucity of data regarding the efficacy of PD1 inhibitors and the effect of CARs on overall survival from Southeast Asia. A retrospective study of patients in the National Cancer Centre Singapore who were diagnosed with melanoma between 2015 and 2020 was conducted. Patients were included in the study if they had stage IV melanoma (advanced melanoma). Sixty-two patients were included in the study. The median age was 62.5 years and acral melanoma was the commonest subtype. Forty-three patients received PD1 inhibitors. Comparing patients who did not receive PD1 inhibitors to patients who received PD1 inhibitors, the former had a median overall survival of 6 months (95% CI: 5.07, 6.93), whereas the latter had a median overall survival of 21 months (95% CI: 13.33, 28.67; *p* < 0.001) (Hazard ratio 0.32; 95% CI: 0.16, 0.63; *p* = 0.001). Amongst patients who received PD1 inhibitors, patients who developed CARs had a greater median overall survival of 33 months (95% CI: 17.27, 48.73) compared to 15 months (95% CI: 9.20, 20.80; *p* = 0.013) for patients who did not (HR 0.29; 95% CI: 0.098, 0.834; *p* = 0.022). This study provides insight into the outcomes of metastatic melanoma in Singapore, and adds to the body of evidence supporting the use of PD1 inhibitors in Asians.

## 1. Introduction

Metastatic melanoma is associated with poor prognosis and high mortality [1]. The development of immune checkpoint inhibitors (ICIs) targeting programmed cell death-1 (PD1) pathways has changed the landscape of melanoma therapy [2]. By blocking the inhibitory pathway between T lymphocytes and antigen-presenting cells or tumour cells, ICIs restore the immune response of effector T cells to tumour cells [2]. Nivolumab and pembrolizumab are examples of ICIs that bind PD1 on T cells, inhibiting the binding of PD1 to its ligand on tumour cells [2].

Multiple large-scale phase III trials studying the impact of PD1 inhibitors on metastatic melanoma outcomes have shown favourable results, but were conducted primarily in white populations [3,4,5]. As the spectrum of melanoma differs between Asian and white populations, it is important to investigate if these findings can be extended to Asian populations. However, there are limited data on the efficacy of PD1 inhibitors in Asian melanoma populations, with few studies on advanced melanoma conducted in Japan and China [6,7,8,9,10]. At present, there are no studies on PD1 inhibitor outcomes for metastatic melanoma in Singapore or Southeast Asia, where Singapore is located.

The use of immune checkpoint inhibitors can result in the development of immune-related adverse events. The development of cutaneous adverse reactions (CARs) was associated with increased overall survival in many studies of predominantly white patients in Europe [11,12,13,14], Canada [15] and America [16]. On the other hand, the impact of CARs on overall survival in Asian populations is less clear, with reports of CARs being associated with increased [6,17,18,19] or decreased [20] overall survival, with multiple reports showing inconclusive data [21,22]. Of note, there is a paucity of data from Southeast Asia.

The aims of this study are two-fold: to investigate the impact of anti-PD1 inhibitors on overall survival in metastatic melanoma, and to describe the cutaneous adverse reactions to anti-PD1 inhibitors in Singapore melanoma patients and their effect on overall survival.

## 2. Materials and Methods

A total of 195 patients with melanoma diagnosed between 2015 and 2021 at the National Cancer Centre Singapore (NCCS) were retrospectively analysed. Consent was obtained from the Institutional Review Board of the NCCS (CIRB: 2018/3065). The details of the patients’ baseline demographics, clinical presentation, treatment and survival data were collected.

Staging was carried out according to the American Joint Committee on Cancer (AJCC) staging system (8th Edition) [23]. *BRAF* and *cKIT* mutations were analysed using next generation sequencing. Overall survival (OS) was computed from the date of diagnosis of stage IV melanoma to the date of demise or last follow-up (for surviving patients). Alive or lost to follow-up patients were censored at the last follow-up date. The median survival time was analysed using the Kaplan–Meier method and differences in survival curves between groups of patients were compared using the log-rank test. A univariate analysis of the association between prognostic factors and survival was performed using the Cox proportional hazard model. For continuous variables, the Mann–Whitney U test was used to compare the medians between the two groups. Counts and percentages were reported for categorical variables, and the chi-squared test or Fisher’s exact test (when more than 20% of the cells had expected frequencies < 5) were used to test for differences between groups. A 2-sided *p*-value < 0.05 was considered statistically significant. Statistical analyses were performed using the IBM SPSS Statistics software version 28.

## 3. Results

### 3.1. Patient Demographics

A total of 62 patients with stage IV melanoma diagnosed between 2015 and 2021 at the National Cancer Centre Singapore (NCCS) were identified (Table 1). Patients who were diagnosed at stage IV or who subsequently progressed to stage IV disease were included in the study. The median age was 62.5 years. There was a predominance of Chinese (74.2%) followed by Malay patients (9.7%), reflecting the ethnic composition of Singapore. Acral melanoma was the commonest subtype seen (38.7%), followed by cutaneous (33.9%) and mucosal melanoma (27.4%). Further details of each subtype can be found in Supplementary Tables S1 and S2. Most patients had metastases in non-central nervous system (CNS) visceral organs (74.2%). A subset of patients opted to undergo testing for *BRAF* and/or *cKIT* mutations. Amongst the patients who were tested for *BRAF* or *cKIT* mutations, 28% had *BRAF* mutations and 19.5% had *cKIT* mutations.

Forty-three patients received treatment with PD1 inhibitors, either pembrolizumab (22.6%), nivolumab (24.2%) or both (22.6%). Nineteen patients did not receive PD1 inhibitors; these patients were given the best supportive care (seven patients), dabrafenib with trametinib (four patients), imatinib (three patients), other systemic therapies (three patients) or radiotherapy (three patients) alone or in combination with other previously mentioned therapies. For the entire cohort of 62 patients, the median overall survival was 10 months, and the median follow-up time was 9.5 months (Table 1).

### 3.2. Survival Analysis

**Figure 1:**
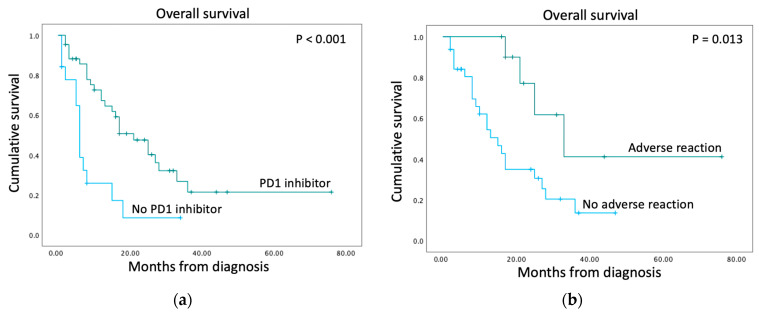
Kaplan–Meier curves for overall survival. (**a**) Patients who received PD1 inhibitors (*n* = 43) had increased overall survival compared to patients who did not (*n* = 19). (**b**) For patients who received PD1 inhibitors, the development of cutaneous adverse reactions (*n* = 11) was associated with increased overall survival compared to no adverse reactions (*n* = 32). *p* values were calculated using the log-rank test.

The prognostic impact of age, gender, ethnicity, melanoma subtype, M category (extent of distant metastasis), *BRAF* mutational status, *cKIT* mutational status, PD1 inhibitor therapy and the use of non-PD1 inhibitor systemic therapies on overall survival was analysed. In univariable analysis, only the use of the PD1 inhibitor was associated with increased overall survival (Table 2). Comparing patients who did not receive PD1 inhibitors to patients who received PD1 inhibitors, the former had a median overall survival of 6 months, whereas the latter had a median overall survival of 21 months (*p* < 0.001, Figure 1a, Table 3). Amongst those who received PD1 inhibitors, 25 patients received PD1 inhibitors alone, and 18 also received non-PD1 therapy. The median overall survival for patients receiving PD1 inhibitors alone was 25 months compared to 17 months for patients who also received other therapies (*p* = 0.197). Although patients who received PD1 inhibitors had a lower median age (60 years) than patients who did not receive PD1 inhibitors (69 years, *p* = 0.008), age was not a significant prognostic factor in the univariate analysis for overall survival (Table 2). In addition, the multivariable analysis of overall survival in patients receiving PD1 inhibitors showed that age was not a significant factor in affecting overall survival, whereas the development of cutaneous adverse reactions was significant (Supplementary Table S3).

### 3.3. Cutaneous Adverse Reactions

There are limited data from Asia regarding the impact of cutaneous adverse reactions on overall survival. In this study, 11 patients who received PD1 inhibitors developed cutaneous adverse reactions whereas 32 patients did not. Comparing the two populations, patients who developed CARs had a greater median overall survival of 33 months (95% CI: 17.27, 48.73) compared to 15 months (95% CI: 9.20, 20.80; *p* = 0.013) for patients who did not develop CARs (HR 0.29; 95% CI: 0.098, 0.834; *p* = 0.022, Table 4, Figure 1b). A comparison of the two groups of patients showed that there was no difference in age, gender, ethnicity, melanoma subtype, M category, *BRAF* mutational status, *cKIT* mutational status and the use of non-PD1 inhibitor systemic therapies before or after PD1-inhibitor treatment (Table 4). Patients who developed CARs were predominantly ethnically Chinese, although this was not significant when compared with patients who did not develop CARs (Table 4).

The commonest cutaneous adverse reaction to PD1 inhibitors was vitiligo (four patients), followed by eczema exacerbation (three patients), lichenoid dermatitis (two patients), psoriasiform eruption (one patient) and exanthem (one patient) (Table 5). One patient had both vitiligo and bullous pemphigoid. Skin biopsies for histological analyses were performed for patients presenting with bullae, lichenoid and psoriasiform eruptions, which all showed dermal eosinophils, supporting a drug-induced cause of the cutaneous manifestations (Table 5). Cutaneous adverse reactions developed following both pembrolizumab and nivolumab use, and in all melanoma subtypes, although there was an over-representation of acral melanoma cases. One patient had to discontinue PD1 inhibitor therapy due to pneumonitis and another patient discontinued PD1 inhibitor therapy due to extensive bullous pemphigoid, which was resolved with the use of topical corticosteroids and oral doxycycline.

## 4. Discussion

Given that the population in Asia has been projected to grow by 44% between 2000 and 2050 [24], the absolute number of melanoma cases can also be expected to increase, highlighting the need for more data on the treatment of melanoma in Asian populations. This study provides insight into the demographics and outcomes of metastatic melanoma in Singapore. Previous studies on melanoma have limited data on stage IV melanoma, and no data on PD1 inhibitor outcomes [25,26,27,28]. In this study, acral melanoma was the commonest subtype, whereas in previous studies, non-acral cutaneous melanoma was commonest when the study population predominantly consisted of stage I-III melanoma and mucosal, ocular and melanoma of unknown primary were included in the study population [26]. In other Singaporean studies of only cutaneous melanoma at predominantly stage I-III, acral lentiginous melanoma was the most common histological subtype [25,28,29]. In studies involving mainly white patients, non-acral cutaneous melanoma was the most common subtype in advanced melanoma [30], reinforcing the differences in melanoma subtypes between ethnicities.

In this study, the median overall survival of patients with PD1 inhibitors was 21 months (median follow-up 9.5 months), which is similar to studies in Japan (OS = 16.93 months, median follow-up unknown) [6] and China (OS = 16 months, median follow-up 11 months) [8]. Mucosal melanoma constituted the highest proportion of patients in the Japanese study [6], whereas acral melanoma was predominant in the Chinese study [8], similar to this study. In the landmark CheckMate 037 study, which consisted primarily of patients from Europe and America, the median overall survival of patients on nivolumab was 15.7 months with a median follow-up of 2 years, but the melanoma subtypes were not stated [5]. In terms of the long-term follow-up of results, the CheckMate 067 trial, which also consisted primarily of white patients from Europe and America, followed 945 patients for at least 5 years, and the median overall survival was more than 60 months for the nivolumab with ipilimumab group [3]. A small Japanese study of 24 patients with mainly acral melanoma treated with nivolumab showed an overall survival of 32.9 months with a median follow-up of 32.9 months and a longer overall survival than other Asian studies; this could be attributed to the longer follow-up time [7]. Larger studies of Asian melanoma patients with longer follow-up durations are required to compare longer-term results with PD1 inhibitors between Asian and white populations.

A subset of patients in this study developed cutaneous adverse reactions to PD1 inhibitors, and this was associated with an increased overall survival, similar to findings from a study in China [18] and some studies from Japan [6,17,19], but differing from other studies from Japan and Taiwan [20,22,31]. Larger studies are required for the generalisation to other Asian populations.

The pathophysiology underlying the association between CARs and overall survival has not been completely elucidated. There are several postulated mechanisms to explain the association between CARs due to PD1 inhibitors and overall survival. One possible mechanism involves shared antigens between melanocytes and melanoma cells, such as tyrosinase and related proteins TRP-1 and TRP-2, gp100 and Melan-A [32]. It is possible that the development of vitiligo indicates an immune reaction towards both normal melanocytes and melanoma cells following PD1 inhibitor administration. In addition, it has been demonstrated that PD1 blockade overcomes T cell exhaustion, leading to the activation of the T cell population both in the tumour microenvironment and systemically [33,34]. As eczema, psoriasis, lichenoid eruptions and vitiligo are T-cell-mediated processes [35], the development of such cutaneous reactions might indicate a robust anti-tumour response and thus improved outcomes.

A previous population-based epidemiological study showed that mucosal melanoma from different anatomical sites exhibited different survival outcomes, with localised pharyngeal, gastroesophageal and vaginal mucosal melanoma exhibiting outcomes that were as poor as metastatic disease, despite aggressive local therapy [36]. Our study included 17 cases of mucosal melanoma, which consisted of melanoma of the anorectum (4 cases), head and neck (6 cases), oesophagus (1 case) and vagina/vulva (6 cases) (Supplementary Table S1). Another study of mucosal melanoma in Chinese patients found genetic variation in mucosal melanoma samples across different body sites, with a reduced overall survival in oesophagus and small bowel melanoma cases [37]. The small number of cases in each site in our study precludes a similar analysis, which would be beneficial in future studies.

Our study has limitations. This was a retrospective study with inherent limitations of incomplete data collection, especially with regard to other epidemiological risk factors such as family history and lifetime sun exposure. Nine cases were lost to follow-up. The number of cases in our study was small, but this is a reflection of the low incidence of melanoma in our population.

## 5. Conclusions

In conclusion, this study adds to the growing body of evidence on the benefit of PD1 inhibitors in Asians. Our study also demonstrates that the development of cutaneous adverse reactions can be associated with an increased overall survival in some patients with metastatic melanoma. Future mechanistic studies would be helpful to tease out the specific pathophysiology underlying this association and aid the prognostication of response to PD1 inhibitors.

## Figures and Tables

**Table 1:** Stage IV melanoma population characteristics (*n* = 62).

Characteristics	Number (%)
Median age (range), years	62.5 (30–86)
Gender	
Female	39 (62.9)
Male	23 (37.1)
Ethnicity	
Chinese	46 (74.2)
Malay	6 (9.7)
Indian	2 (3.2)
White	3 (4.8)
Others	5 (8.1)
Subtype	
Acral	24 (38.7)
Cutaneous	21 (33.9)
Mucosal	17 (27.4)
M category	
1a	11 (17.7)
1b	16 (25.8)
1c	30 (48.4)
1d	5 (8.1)
*BRAF* status ^a^	
Wild type	36 (72)
Mutation	14 (28)
*cKIT* status ^b^	
Wild type	33 (80.5)
Mutation	8 (19.5)
PD1 inhibitor	
Pembrolizumab	14 (22.6)
Nivolumab	15 (24.2)
Both	14 (22.6)
None	19 (30.6)
Any administration of systemic non-PD1 therapy	
No	34 (54.8)
Yes	28 (45.2)
Dabrafenib/Trametinib	8
Imatinib	6
Clinical Trial drug	5
Paclitaxel/Carboplatin	4
Temozolamide	2
Decarbazine	1
Vemurafenib	1
Encorafenib/Binimetinib	1
Overall survival (range), months	10 (1–76)
Follow-up time (range), months	9.5 (1–75)

^a^ A total of 12 patients were not tested for *BRAF* mutations. ^b^ A total of 21 patients were not tested for *cKIT* mutations.

**Table 2:** Univariate analysis for overall survival in stage IV melanoma.

Characteristics	E/N	Hazard Ratio (95% CI)	*p*-Value
Age at diagnosis			
<60 years old	12/22	1	ref
≥60 years old	28/40	1.74 (0.87, 3.47)	0.119
Gender			
Female	24/39	1	ref
Male	16/23	0.86 (0.64, 1.22)	0.450
Ethnicity			
Chinese	31/46	1	ref
Malay	4/6	1.10 (0.36, 3.13)	0.862
Indian	2/2	1.02 (0.24, 4.34)	0.983
White	2/3	0.72 (0.17, 3.03)	0.649
Others	1/5	1.73 (0.22, 13.69)	0.605
Subtype			
Acral	14/24	1	ref
Cutaneous	16/21	1.61 (0.78, 3.32)	0.195
Mucosal	10/17	1.13 (0.50, 2.56)	0.762
M category			
1a	6/11	1	ref
1b	9/16	0.82 (0.29, 2.33)	0.704
1c	22/30	2.10 (0.85, 5.23)	0.110
1d	3/5	1.48 (0.36, 6.05)	0.586
*BRAF* status			
Wild type	24/36	1	ref
Mutation	9/14	1.72 (0.78, 3.78)	0.176
*cKIT* status			
Wild type	21/33	1	ref
Mutation	6/8	1.45 (0.57, 3.71)	0.436
PD1 inhibitor			
No	14/19	1	ref
Yes	26/43	0.32 (0.16, 0.63)	0.001
Any administration of systemic non-PD1 therapy			
No	18/34	1	ref
Yes	22/28	1.55 (0.82, 2.95)	0.182

CI: Confidence interval. *p* value calculated using the Cox proportional hazards model. E/N: Events/Number of cases. Ref: reference.

**Table 3:** Comparing patients who received PD1 vs. no PD1 inhibitor.

Characteristics	No PD1 (*n* = 19)	PD1 (*n* = 43)	*p*-Value
Median age (range), years	69 (30–86)	60 (31–76)	0.008 ^a^
Gender			
Female	10 (52.6)	29 (67.4)	0.266 ^b^
Male	9 (47.4)	14 (32.6)	
Ethnicity			
Chinese	14 (73.7)	32 (74.4)	0.186 ^c^
Malay	0 (0)	6 (14.0)	
Indian	1 (5.3)	1 (2.3)	
White	2 (10.5)	1 (2.3)	
Others	2 (10.5)	3 (7.0)	
Subtype			
Acral	8 (42.1)	16 (37.2)	0.830 ^c^
Cutaneous	7 (36.8)	14 (32.6)	
Mucosal	4 (21.1)	13 (30.2)	
M category			
1a	2 (10.5)	9 (20.9)	0.570 ^c^
1b	4 (21.1)	12 (27.9)	
1c	12 (63.2)	18 (41.9)	
1d	1 (5.3)	4 (9.3)	
*BRAF* status			
Wild type	9 (60.0)	27 (77.1)	0.304 ^c^
Mutation	6 (40.0)	8 (22.9)	
*cKIT* status			
Wild type	7 (70.0)	26 (83.9)	0.378 ^c^
Mutation	3 (30.0)	5 (16.1)	
Any administration of systemic non-PD1 therapy			
No	9 (47.7)	25 (58.1)	0.432 ^b^
Yes	10 (52.6)	18(41.9)	
Median overall survival, months (95% CI)	6 (5.07, 6.93)	21 (13.33, 28.67)	<0.001 ^d^

^a^ *p*-value calculated using Mann–Whitney U test. ^b^ *p*-value estimated using chi-squared test. ^c^ *p*-value estimated using Fisher’s exact test. ^d^ *p*-value estimated using log-rank test. CI, confidence interval.

**Table 4:** Comparing patients with cutaneous adverse reactions vs. no cutaneous adverse reactions to PD1 inhibitors.

Characteristics	No CAR (*n* = 32)	CAR (*n* = 11)	*p*-Value
Median age (range)	60.5 (31–76)	58 (33–74)	0.666 ^a^
Gender			
Female	23 (71.9)	6 (54.5)	0.457 ^b^
Male	9 (28.1)	5 (45.5)	
Ethnicity			
Chinese	22 (68.8)	10 (90.9)	0.757 ^b^
Malay	5 (15.6)	1 (9.1)	
Indian	1 (3.1)	0 (0)	
White	1 (3.1)	0 (0)	
Others	3 (9.4)	0 (0)	
Subtype			
Acral	9 (28.1)	7 (63.6)	0.079 ^b^
Cutaneous	13 (40.6)	1 (9.1)	
Mucosal	10 (31.3)	3 (27.3)	
M category			
1a	6 (18.8)	3 (27.2)	0.631 ^b^
1b	8 (25.0)	4 (36.4)	
1c	14 (43.8)	4 (36.4)	
1d	4 (12.5)	0 (0)	
*BRAF* status			
Wild type	19 (70.4)	8 (100)	0.154 ^b^
Mutation	8 (29.6)	0 (0)	
*cKIT* status			
Wild type	18 (85.7)	8 (80.0)	0.999 ^b^
Mutation	3 (14.3)	2 (20.0)	
Any administration of systemic non-PD1 therapy			
No	17 (53.1)	8 (72.7)	0.309 ^b^
Yes	15 (46.9)	3 (27.3)	
PD1 inhibitor line of treatment			
First line	24 (75)	9 (81.8)	0.999 ^b^
Second line or later	8 (25)	2 (18.2)	
Median overall survival, months (95% CI)	15 (9.20, 20.80)	33 (17.27, 48.73)	0.013 ^c^

CAR: Cutaneous adverse reaction. CI, confidence interval. ^a^ *p*-value calculated using Mann–Whitney U test. ^b^ *p*-value estimated using Fisher’s exact test. ^c^ *p*-value estimated using log-rank test.

**Table 5:** Cutaneous adverse reactions to PD1 inhibitors.

Cutaneous Adverse Reaction	No. of Patients	PD1 Inhibitor	Melanoma Subtype	Histology of Skin Reaction	Management
Vitiligo	3 (27.3%)	Pembrolizumab then nivolumab and ipilimumab	Mucosal	Not performed	Continue PD1 inhibitor
		Nivolumab	Mucosal	Not performed	Continue PD1 inhibitor
		Pembrolizumab	Acral	Not performed	Continue PD1 inhibitor
Vitiligo and bullous pemphigoid (BP)	1 (9.1%)	Nivolumab	Acral	Subepidermal blister with dermal lymphocytes, histiocytes and eosinophils	Topical corticosteroids, oral doxycycline. Stopped PD1 inhibitor (bullous pemphigoid)
Eczema exacerbation	3 (27.3%)	Pembrolizumab then nivolumab and ipilimumab	Cutaneous	Not performed	Topical corticosteroids, continue PD1 inhibitor
		Nivolumab	Acral	Not performed	Topical corticosteroids, continue PD1 inhibitor
		Nivolumab then pembrolizumab	Acral	Not performed	Topical corticosteroids, continue PD1 inhibitor
Lichenoid dermatitis	2 (18.1%)	Pembrolizumab then nivolumab	Acral	Interface dermatitis with subcorneal neutrophilic collections and perivascular dermal lymphocytic infiltrate with plasma cells and eosinophils	Topical corticosteroids, continue PD1 inhibitor
		Nivolumab	Acral	Irregular acanthosis and spongiosis with superficial dermal oedema and chronic inflammatory infiltrate with eosinophils	Topical corticosteroids, continue PD1 inhibitor
Psoriasiform eruption	1 (9.1%)	Nivolumab	Acral	Irregular psoriasiform hyperplasia with focal mild spongiosis where a small collection of neutrophils is seen in the upper epidermis. Superficial perivascular infiltrate of lymphocytes and eosinophils	Topical corticosteroids, stopped PD1 inhibitor (pneumonitis)
Exanthem	1 (9.1%)	Nivolumab	Mucosal	Not performed	Topical corticosteroids, continue PD1 inhibitor

## Data Availability

The original contributions presented in the study are included in the article/Supplementary Materials; further inquiries can be directed to the corresponding authors.

## Supplementary Materials

The following supporting information can be downloaded at: https://www.mdpi.com/article/10.3390/diagnostics14151601/s1, Table S1: Sites of melanoma; Table S2: Histological details of cutaneous melanoma cases; Table S3: Analysis of factors affecting overall survival in patients receiving PD1 inhibitors using the multivariable Cox proportional hazard regression model (*n* = 43).
