# Supplementary material for: Cutaneous Adverse Reactions and Survival Outcomes of Advanced Melanoma Treated with Immune Checkpoint Inhibitors in an Academic Medical Centre in Singapore
Source: Diagnostics (Basel). 2024 Jul 25;14(15):1601. doi: 10.3390/diagnostics14151601 (PMC11311877; doi:10.3390/diagnostics14151601)
Supplement: Supplementary file 1 [file diagnostics-14-01601-s001.zip › diagnostics-3071324-supplementary.pdf]

Supplementary Table S1. Sites of melanoma.

Subtype	Location	Number of patients
Acral	Finger	1
Acral	Foot	17
Acral	Toe	6
Cutaneous	Limbs (excluding finger, toe, hand, foot)	7
Cutaneous	Head and neck	3
Cutaneous	Trunk	11
Mucosal	Anorectum	4
Mucosal	Head and neck	6
Mucosal	Oesophagus	1
Mucosal	Vagina/vulva	6

Supplementary Table S2. Histological details of cutaneous melanoma cases.

Patient	Pattern	Cell type
1	Unknown	Spindle
2	Nodular	Epithelioid & Spindle
3	Unknown	Epithelioid
4	Nodular	Epithelioid
5	Superficial spreading	Epithelioid
6	Unknown	Epithelioid
7	Unknown	Unknown
8	Nodular	Epithelioid
9	Unknown	Epithelioid
10	Unknown	Epithelioid
11	Nodular	Epithelioid
12	Unknown	Epithelioid
13	Nodular	Unknown
14	Nodular	Epithelioid
15	Nodular	Epithelioid
16	Unknown	Unknown
17	Nodular	Epithelioid
18	Pagetoid and Lentiginous	Epithelioid
19	Unknown	Epithelioid
20	Unknown	Unknown
21	Unknown	Epithelioid

Supplementary Table S3. Analysis of factors affecting overall survival in patients receiving PD1 inhibitors using the multivariable Cox proportional hazard regression model (n=43)

**Characteristics**	**Hazard Ratio (95% CI)**	**P-value**
Age at diagnosis	1.00 (0.96, 1.04)	0.96
Gender		
Female	1	ref
Male	2.33 (0.74, 7.29)	0.15
Ethnicity		
Chinese	1	ref
Malay	1.04 (0.25, 4.32)	0.96
Indian	0.11 (0.01, 1.56)	0.11
White	0.00 (0.00, 0.00)	0.97
Others	0.00 (0.00, 0.00)	0.99
Subtype		
Acral	1	ref
Cutaneous	2.59 (0.75, 8.95)	0.13
Mucosal	1.03 (0.27, 3.89)	0.96
Cutaneous adverse reaction		
No	1	ref
Yes	0.20 (0.06, 0.68)	**0.01**

CI, confidence interval
